# Structural and Phase Stability and Deuterium Sorption Behavior of Be_12_Ti Titanium Beryllide Under Simulated Ion Irradiation

**DOI:** 10.3390/ma19173755

**Published:** 2026-09-03

**Authors:** Alexey Dikov, Yelizaveta Maximkina, Timur Kulsartov, Anna Shmonina, Lyubov Dikova, Boris Ivanov, Saulet Askerbekov, Pavel Oreshkin, Vsevolod Fettsov, Mikhail Podoinikov, Sergey Udartsev

**Affiliations:** 1 Institute of Nuclear Physics, 1 Ibragimov St., 050032 Almaty, Kazakhstan; dikov@inp.kz (A.D.); tima@physics.kz (T.K.);; 2 Department of General Physics, Satbayev University, 22 Satbayev St., 050000 Almaty, Kazakhstan; 3 Ulba Metallurgical Plant, 102 Abay Av., 070005 Ust-Kamenogorsk, Kazakhstan

**Keywords:** titanium beryllide (Be_12_Ti), deuterium, ion irradiation, gas desorption, microstructure, X-ray diffraction

## Abstract

Plasma-facing components of fusion facilities must withstand a wide range of operating loads. Their structural integrity is strongly affected by plasma particles, including deuterium (D), tritium (T), and helium (He), as well as by 14 MeV neutrons, steady-state heat fluxes, and recurrent intense transient thermal loads. In most current studies, the effects of individual operating factors—such as neutron irradiation, plasma exposure, and thermal loading—are investigated separately, allowing the contribution of each factor to changes in material properties to be evaluated. However, degradation mechanisms arising from the synergistic action of these factors require investigation using more sophisticated experimental methodologies. The present study aims to develop a methodology for the simulation testing of materials intended for fusion applications and at investigating the structural and phase stability of Be_12_Ti, as well as its deuterium sorption behavior, following high-energy irradiation with 14 MeV D^+^ ions. Irradiation was performed at ion flux densities of approximately 8.6 × 10^14^ ions·cm^−2^·s^−1^ and 1.06 × 10^20^ ions·cm^−2^·s^−1^. The irradiation-induced target temperatures reached approximately 400K and 1000K, respectively. An intermetallic Be_12_Ti alloy produced at the Ulba Metallurgical Plant in Kazakhstan was used as the target material. The selection of Be_12_Ti was motivated by existing fusion reactor concepts that consider this intermetallic compound as a neutron multiplier material, including the Japanese JA DEMO concept.

## 1. Introduction

Fusion energy offers humanity a potential means of maintaining current levels of energy production as hydrocarbon fuels and economically recoverable uranium-235 resources become depleted. However, the service life of structural and functional materials remains one of the key unresolved challenges preventing the successful implementation of fusion technologies in the energy sector. Therefore, investigating material behavior under conditions representative of actual fusion reactor operating environments is among the most pressing challenges in modern radiation materials science.

The synergistic effects of thermal, plasma, and neutron loads initiate various degradation processes, including embrittlement, transmutation, sputtering, flaking and spallation, evaporation, and cracking [1]. These processes result in plasma contamination by ionized atoms originating from plasma-facing materials, thereby increasing the overall bremsstrahlung losses and reducing the plasma temperature required to sustain fusion reactions [2]. Because the intensity of bremsstrahlung radiation is proportional to the square of the ionic charge, beryllium is considered a suitable low-atomic-number element for first-wall applications. Owing to its low atomic number, beryllium impurities entering the plasma cause substantially lower radiative losses than impurities originating from higher-Z materials. An additional advantage of beryllium is its ability to contribute to neutron multiplication and tritium-breeding processes required to sustain the fusion fuel cycle [3,4,5]. These properties make beryllium a promising constituent of blanket materials for advanced fusion reactors.

The principal disadvantages of pure metallic beryllium include its carcinogenicity, susceptibility to radiation-induced swelling, and relatively low melting temperature compared with those of its intermetallic compounds. For these reasons, several fusion reactor concepts consider beryllium compounds, including Be_22_V and Be_12_Ti, as candidate materials for plasma-facing and blanket applications. Among these compounds, vanadium beryllide exhibits a higher atomic density; however, its tritium-breeding efficiency is approximately 30–40% lower than that of titanium beryllide [6]. The distribution and retention of tritium within a material are not necessarily correlated with a high atomic density [3]. Therefore, among the two promising beryllide compounds, titanium beryllide may provide a more advantageous combination of properties for blanket applications.

The binding energy of hydrogen and tritium atoms at vacancies and other lattice trapping sites in Be_12_Ti is approximately 0.4 eV lower than that in pure beryllium [7]. Consequently, tritium can be released from Be_12_Ti more readily at blanket operating temperatures, facilitating its extraction and return to the fuel cycle. At the same time, the amount of tritium retained or accumulated within reactor components is strictly limited by safety requirements. For example, the tritium inventory limit for ITER is approximately 700–1000 g [8]. Exceeding this limit would require reactor operation to be interrupted for tritium removal and component cleaning. Moreover, sophisticated tritium-breeding systems must be incorporated into fusion reactor designs to compensate for fuel losses caused by retention in structural and functional materials. Therefore, from the perspective of fuel-cycle efficiency and process predictability, the ability of beryllide materials to support tritium breeding while facilitating its subsequent release represents an important advantage.

In addition to exposure to plasma particles, fusion reactor materials will be subjected to intense irradiation by neutrons with energies of approximately 14 MeV, accompanied by quasi-steady-state thermal loads at temperatures of 600–900 °C and transient high-intensity heat loads [9,10]. Under ITER operating conditions, the plasma density is expected to reach approximately 10^20^ particles·m^−3^. Despite the use of magnetic coils to stabilize the plasma shape and position, interactions between the plasma and blanket, first-wall, and divertor materials cannot be completely eliminated. In particular, edge-localized modes (ELMs) may occur during high-confinement-mode operation [10], together with plasma instabilities, disruptions, and vertical displacement events [1]. These phenomena produce transient plasma bursts that deposit substantial amounts of energy onto the divertor and first-wall surfaces.

For the ITER tokamak, the steady-state heat flux incident on divertor materials is expected to be approximately 20 MW·m^−2^ [1,10], whereas the transient heat flux during ELM events may reach approximately 1 GW·m^−2^ [11]. Even more extreme conditions may arise during plasma disruptions and vertical displacement events, when the deposited energy density can reach up to 60 MJ·m^−2^ over a period of 100–300 ms [12]. Such loads can cause surface melting, severe erosion, and intense sputtering of divertor materials.

Material degradation, however, is not only determined by direct plasma-induced surface damage. Charged particles, including D, T, and He, are primarily implanted into near-surface layers. Nevertheless, at elevated temperatures and over prolonged operating periods, implanted species may migrate into deeper regions of the material. In combination with irradiation-induced defects, this process promotes the formation of hydrogen-trapping sites, gas accumulation, and the development of internal stresses. Consequently, the assessment of material radiation resistance should account not only for surface erosion but also for thermally activated processes governing gas accumulation, retention, migration, and desorption from the bulk of the material.

To date, numerous experiments have investigated individual degradation mechanisms in different materials under conditions designed to approximate those expected in fusion reactors [13]. However, experimental studies capable of assessing material stability under the simultaneous and synergistic action of irradiation, plasma exposure, and thermal loading remain limited. Accordingly, the present study focuses on the intermetallic compound Be_12_Ti, which is considered a promising candidate material for fusion reactor applications.

Irradiation with 14 MeV deuterium ions was employed as an experimentally accessible simulation approach capable of simultaneously producing irradiation- and thermally induced defects within the material bulk and enabling an assessment of hydrogen-isotope accumulation and retention. This approach provides insight into the key mechanisms governing the degradation of Be_12_Ti under combined exposure to high-energy particles and elevated temperatures.

## 2. Materials and Methods

**Figure 1:**
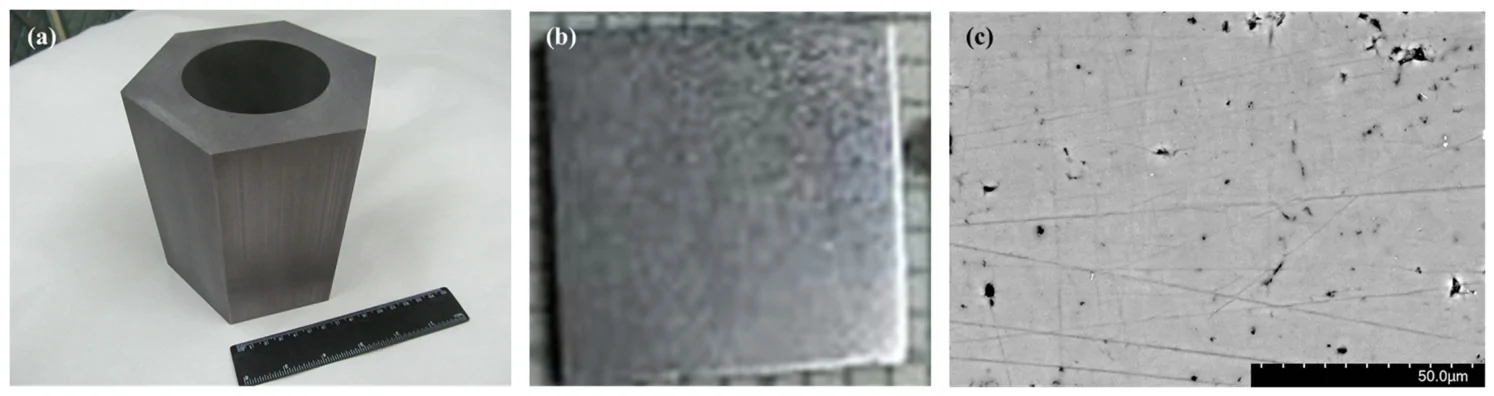
(**a**) Be_12_Ti product; (**b**) experimental specimen; (**c**) specimen surface after the mechanical processing.

The material selected for this study was the Be_12_Ti intermetallic compound produced by vacuum hot pressing of powders at Ulba Metallurgical Plant JSC, Ust-Kamenogorsk, Kazakhstan. The particle size of the starting powder used for sintering was up to 25 µm. Ulba Metallurgical Plant JSC has developed an industrial-scale technology for manufacturing Be_12_Ti components (Figure 1a) with a natural isotopic composition comprising ^9^Be and the naturally occurring titanium isotopes ^46^Ti, ^47^Ti, ^48^Ti, ^49^Ti, and ^50^Ti [14]. According to the material certificate, the manufactured material was single-phase and had a density of 2.26 g·cm^−3^. Its nominal elemental composition was 92.3 at.% Be and 7.69 at.% Ti, with the total impurity content not exceeding 0.01%.

The experiments were performed using square plate specimens (Figure 1b) with dimensions of 5 × 5 × 2 mm^3^. The specimens were cut from bulk blocks by electrical discharge machining. Prior to testing, the specimen surfaces were mechanically ground using abrasive papers of progressively finer grit and subsequently polished on a polishing cloth with a chromium oxide-based polishing compound.

Microstructural characterization was performed using a Hitachi TM4000Plus scanning electron microscope equipped with a Bruker QUANTAX 75 energy-dispersive X-ray spectroscopy system (manufactured by Bruker Corporation, Bruker, Germany) for elemental analysis. Figure 1c shows the specimen surface after mechanical preparation. The micrograph reveals polishing marks and residual porosity manifested as discontinuities and voids within the material. The grain structure could not be clearly distinguished under the applied imaging conditions.

X-ray diffraction measurements and phase analysis were performed using a D8 ADVANCE diffractometer (manufactured by Bruker Corporation, Bruker, Germany) in a Bragg–Brentano θ-θ configuration. A copper-anode X-ray tube with a wavelength of 1.5406 Å was operated at 40 kV and 40 mA. The diffractometer was equipped with a NaI scintillation detector. Diffraction patterns were recorded over a 2θ range of 15–90° with a step size of 0.02°. The counting time was 2 s per step for the specimens before ion irradiation and 3s per step for the irradiated specimens. During data acquisition, the specimen holder was rotated at 60 rpm using the built-in Adjust control software. Qualitative phase identification was performed using EVA software and the JCPDS/PDF-2 reference database.

The sorption and retention of hydrogen isotopes following irradiation were evaluated by thermal desorption spectroscopy (TDS) in a vacuum chamber at a constant heating rate. During heating under vacuum, gaseous species were released from the specimen and detected using a mass spectrometer. Before the TDS measurement series, the experimental system was subjected to a technical bakeout, followed by a blank TDS run without a specimen, while reproducing the specimen-loading procedure. The system bakeout was performed under continuous evacuation with a scroll pump by heating the chamber to 900 °C at a rate of 10 °C·min^−1^. The residual pressure in the measurement chamber before heating was approximately 10^−6^ Torr.

The TDS procedure was conducted as follows. After a residual pressure of approximately 10^−6^ Torr had been reached, gas-evolution monitoring was initiated, and the specimen was heated linearly to the target temperature at a rate of 10 °C·min^−1^. Upon reaching 900 °C, the specimen was held at this temperature for 5 min. It was then cooled at a rate of 20 °C·min^−1^. A detailed description of the experimental setup and the TDS methodology is provided in Ref. [4].

### Experimental Setup and Irradiation Conditions

In the present experiment, 14 MeV deuterium ions were selected as light-ion projectiles to simulate selected effects of neutron irradiation. The use of ion irradiation was determined by the experimental limitations associated with the direct reproduction of fusion reactor conditions [15]. Generating an intense neutron flux at an energy of approximately 14 MeV remains technically challenging, whereas irradiation in fast reactors requires long exposure times to achieve the required fluence [16]. Consequently, ion beams are widely employed to investigate radiation-induced defect formation in candidate materials for fusion applications [17,18].

Although ion irradiation cannot fully reproduce neutron-induced damage, it enables the controlled generation of defects and facilitates investigation of the implantation, retention, and release of hydrogen isotopes. In this study, irradiation with 14 MeV D^+^ ions was employed because this energy provides substantially greater deuterium penetration depths than low-energy plasma exposure and therefore enables the assessment of bulk deuterium accumulation and gas-release processes in Be_12_Ti [16,18,19].

The specimens were irradiated using the U-150M isochronous cyclotron at the Institute of Nuclear Physics, Almaty, Kazakhstan. The cyclotron is capable of accelerating protons, deuterons, α particles, and ^3^He^2+^ ions, with adjustable final beam energies. The hydrogen-ion flux density can be controlled by varying the beam current from 2 to 30 µA. The irradiation station is equipped with a specimen-cooling system capable of removing thermal power of up to 12 kW, thereby allowing the use of high-intensity ion beams.

Based on the accelerator capabilities, two irradiation regimes were selected: stationary ion flow (SIF), intended to simulate the operating conditions of first-wall materials during steady-state plasma operation, and combined irradiation (CI), designed to reproduce the transition between high-intensity transient and steady-state loading conditions.

For the SIF regime, the specimens were irradiated with 14 MeV deuterium ions at a beam current of 2.7 µA. The ion flux density under these conditions was moderately high and amounted to 1.06 × 10^14^ ions·cm^−2^·s^−1^. Under such irradiation conditions, irradiation-induced defect generation, ion implantation, and thermally and radiation-enhanced diffusion occur simultaneously. Surface blistering and ion-induced sputtering or erosion may also take place. To accumulate a relatively high irradiation dose, the exposure duration was 11 h. The resulting ion fluence was 4.2 × 10^18^ ions·cm^−2^.

The CI regime was implemented in two consecutive stages. During the first stage, the specimens were irradiated with 14 MeV deuterium ions at a beam current of 20 µA for 22 min, corresponding to an ion flux density of 7.8 × 10^14^ ions·cm^−2^·s^−1^. The beam current was then reduced to 2 µA, corresponding to an ion flux density of 0.7 × 10^14^ ions·cm^−2^·s^−1^, and irradiation was continued for an additional 60 min.

This two-stage regime was designed to simulate an abrupt change in plasma loading, such as a transition from a high-intensity plasma disruption or edge-localized-mode event to stable steady-state plasma operation. It represents a situation in which a first-wall component is initially exposed to an intense particle flux, for example, during a transient event or reactor power ramp-up, followed by stabilization of the plasma and transition to normal operating conditions.

The ion flux densities and accumulated fluences corresponding to the two irradiation regimes are summarized in Table 1.

**Figure 2:**
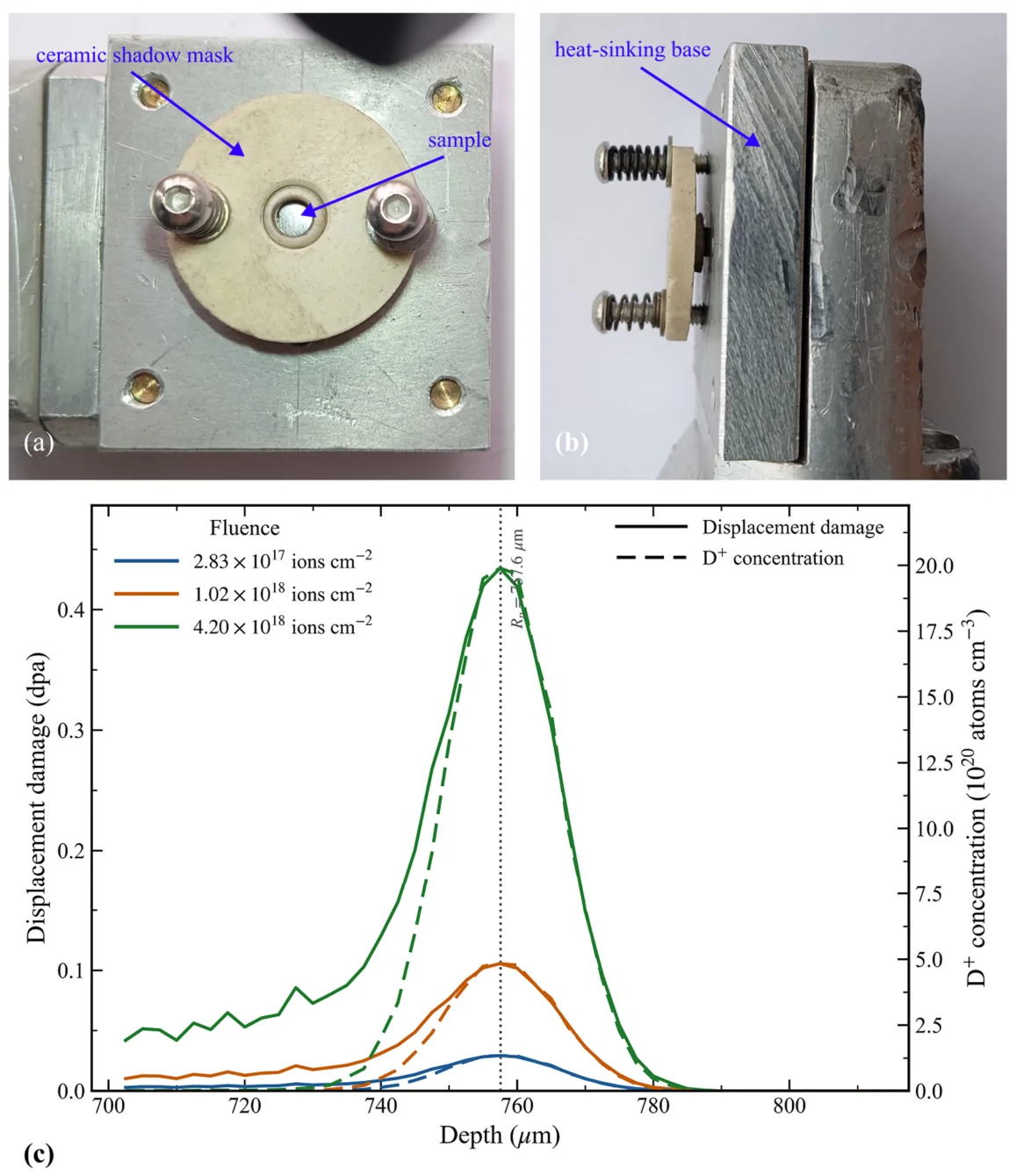
General view of the irradiation target and SRIM simulation results: (**a**) top view of the target; (**b**) side view of the target; (**c**) displacement damage & D^+^ concentration plots.

During irradiation, the specimens were mounted on an aluminum holder that provided continuous heat removal. Each specimen was pressed against the heat-sink plate using a ceramic washer made of high-density technical-grade corundum (Figure 2a,b). The ceramic washer also served as an ion-beam mask, shielding ions that did not impinge directly on the specimen and preventing their contribution to the measured beam current.

Before mounting, the specimens and the ceramic washer were cleaned with ethyl alcohol. A graphite collimator was used to focus and shape the ion beam. Beam-focusing stability was monitored visually throughout irradiation using an industrial television imaging system.

The number of ions incident on the target was determined from the measured beam current using an ORTEC Model 439 (manufactured by AMETEC ORTEC, Oak Ridge, TN, USA) current integrator.

Prior to irradiation, the penetration depth of deuterium ions in Be_12_Ti was calculated using SRIM 2013. According to the simulation results, the mean projected range of 14 MeV D^+^ ions was approximately 757 µm (Figure 2c). Although the deuterium implantation region was relatively narrow, irradiation-induced heating and the resulting directional heat flux were expected to promote the diffusion of implanted deuterium into the bulk of the material.

The temperature increase resulting from the interaction of accelerated ions with the specimen was evaluated using COMSOL Multiphysics 6.2 with the Heat Transfer in Solids module and the finite element method. The thermal conductivity and heat-capacity parameters of Be_12_Ti used in the calculations were obtained from the IAEA Thermo-Physical Materials Properties Database [20].

A three-dimensional model closely reproducing the experimental target configuration was constructed using the built-in meshing tools. The model included an aluminum substrate acting as a heat sink and a Be_12_Ti specimen with a defined irradiation area corresponding to the region exposed to the ion beam. Because the specimen thickness considerably exceeded the calculated ion range, all ions incident on the target were assumed to be fully stopped within the specimen. For the purposes of the thermal analysis, the contribution of nuclear collisions to energy deposition was considered negligible, and the total energy deposited in the specimen during irradiation was assumed to be converted into heat.

**Figure 3:**
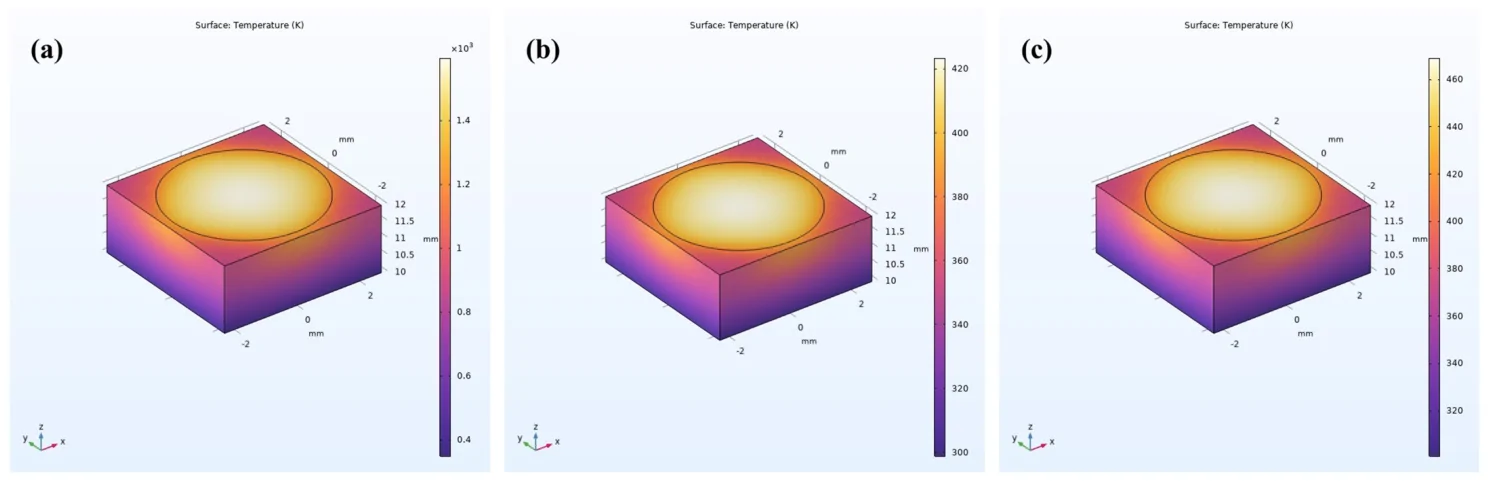
Simulated heating of the Be_12_Ti specimen under the specified ion-irradiation conditions: (**a**) CI at a beam current of 20 µA for 22 min; (**b**) CI at a beam current of 2 µA for 60 min; and (**c**) SIF at a beam current of 2.7 µA for 11 h.

The simulations were performed for the two irradiation regimes described above. Figure 3 presents the simulated temperature distribution and heat-flow direction in the Be_12_Ti specimen under ion irradiation. Under the SIF conditions, the temperature of the irradiated Be_12_Ti surface reached approximately 470 K. During the first stage of the CI regime, the irradiated surface temperature reached approximately 1000 K. After the ion flux density was reduced, the temperature decreased to approximately 420 K. Continuous heat removal through the aluminum substrate produced a directional temperature gradient across the specimen.

## 3. Results and Discussion

### 3.1. Assessment of Irradiation-Induced Changes in the Phase Composition

**Figure 4:**
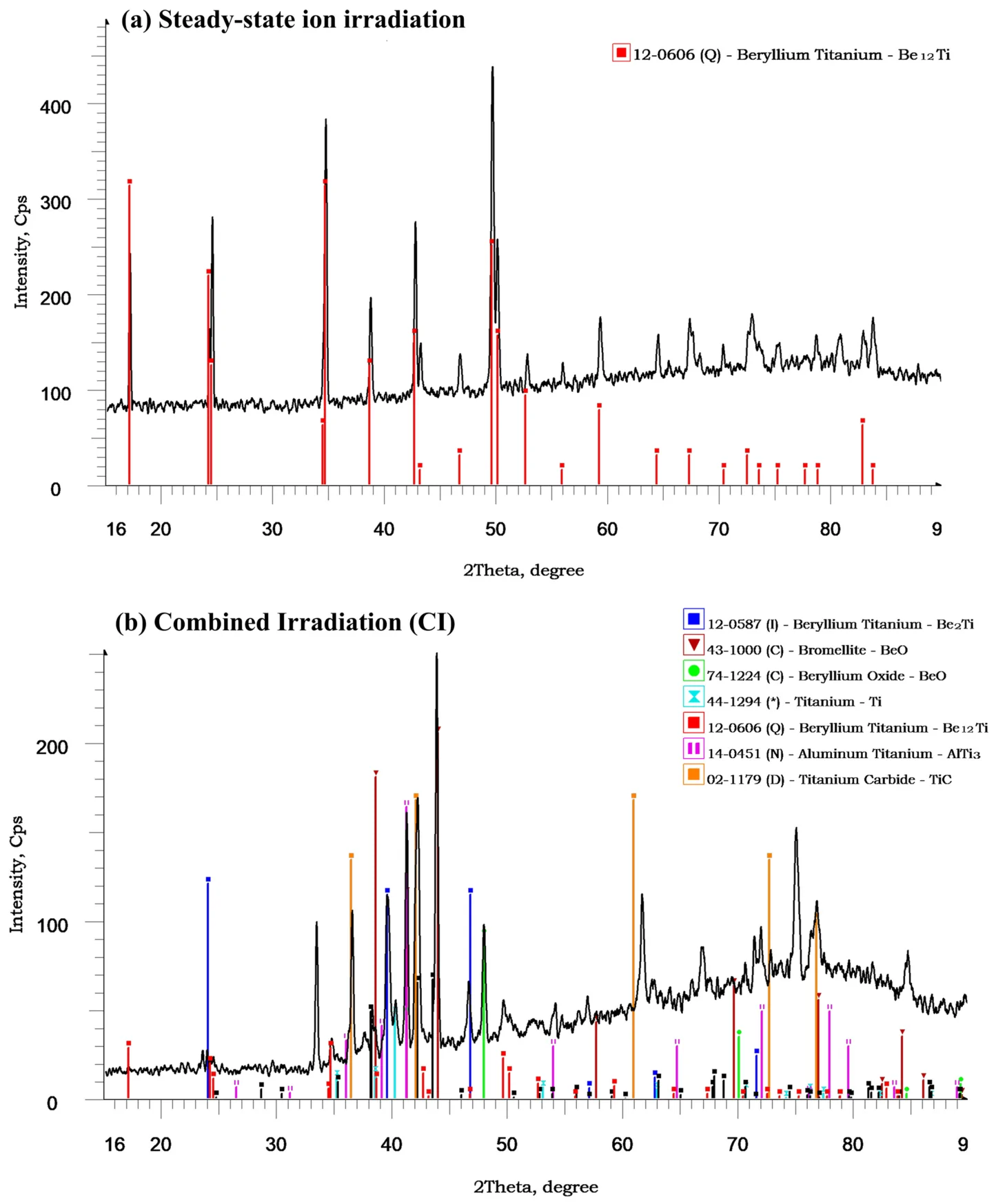
X-ray diffraction patterns of the Be_12_Ti specimens irradiated under the following regimes: (**a**) stationary ion flow (SIF) and (**b**) combined irradiation (CI).

Figure 4 presents the results of the structural characterization of the Be_12_Ti specimens before and after irradiation. The X-ray diffraction pattern of the unirradiated Be_12_Ti specimen (Figure 4a) contains reflections corresponding to the Be_12_Ti phase (JCPDS/PDF card No. 12-0606). Several unidentified diffraction peaks are also observed at 2θ values of approximately 29.5° and 80.5°. The intensity of the most prominent diffraction peak, located at approximately 2θ = 50°, is about 420 counts per second (cps).

Irradiation of Be_12_Ti with deuterium ions under the SIF regime resulted in an increase in the diffuse background intensity (Figure 4a), indicating the disordered nature of long–range atoms in the X-ray amorphous state structure. The formation of the X-ray amorphous halo occurs in the range of angles 30–85°. The intensity of the basic reflexes does not change significantly.

The formation of X-ray amorphous halos under ion irradiation of solids is a well-known fact [21]. Irradiation with high-energy deuterium ions (14 MeV) with a range of up to ~800 microns in Be_12_Ti (see Figure 2c) creates defects throughout the range region, causing lattice destabilization and disordering of the crystal structure, which is manifested in X-ray amorphous halos on diffractograms.

Following deuterium-ion irradiation under the CI regime (Figure 4b), the diffraction pattern also exhibited pronounced disruption of long-range atomic order (broad amorphous halo extending over the 2θ range of 30–85°), together with an almost twofold decrease in the intensities of the Be_12_Ti reflections. In addition, two polymorphic modifications of beryllium oxide were clearly identified: a hexagonal BeO phase corresponding to card № 43-1000 and a face-centered cubic BeO phase corresponding to card № 74-1224. Not all reflections expected for the hexagonal BeO phase were observed, suggesting the development of a preferred crystallographic orientation.

Irradiation under the CI regime also resulted in the formation of elemental titanium with a hexagonal crystal structure, corresponding to card № 44-1294, as well as titanium carbide phases, most of whose reflections were consistent with card № 02-1179. Similar to the BeO crystallites, the titanium carbide crystallites exhibited a pronounced preferred orientation. This conclusion is supported by the absence of the expected titanium carbide reflection at approximately 2θ = 61°. The reflection observed at 61,8° was markedly shifted relative to the reference positions listed in card № 02-1179 and therefore most likely originated from another phase. By contrast, the reflection at 76.87° was again in good agreement with card № 02-1179. Several additional reflections from unidentified phases were also detected in the diffraction pattern of the specimen irradiated under the CI regime.

The observed changes in the phase composition of Be_12_Ti and the development of preferred crystallographic orientations in the newly formed phases can be attributed to the substantial temperature increase caused by the high-intensity ion exposure during the first stage of the CI regime.

### 3.2. Assessment of Hydrogen-Isotope Sorption

**Figure 5:**
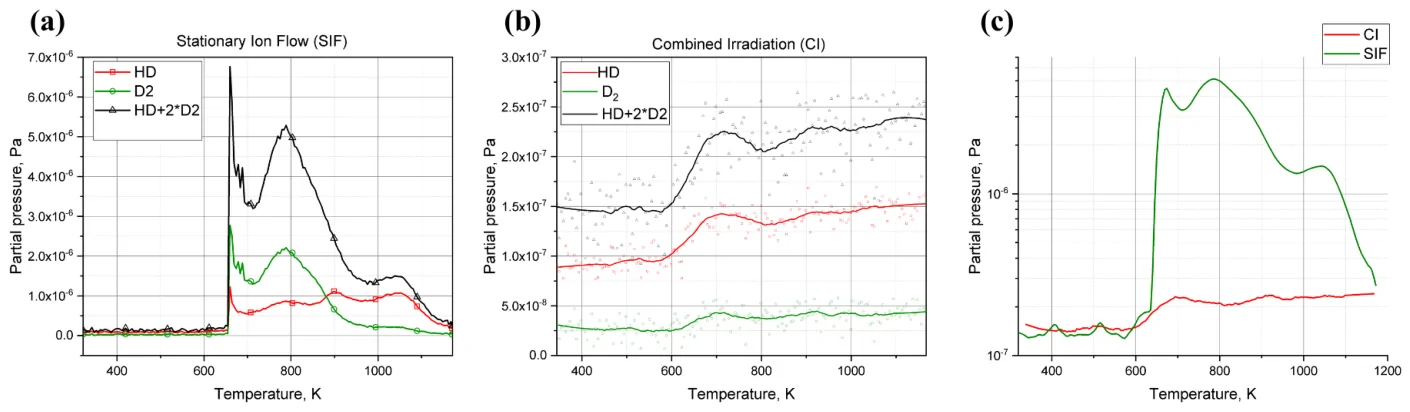
Thermal desorption spectra of the Be_12_Ti specimens: (**a**) specimen irradiated under the stationary ion flow (SIF) regime; (**b**) specimen irradiated under the combined irradiation (CI) regime; and (**c**) integrated gas-release signals.

As shown in Figure 5, the gas-release behavior of the SIF specimen was the most readily interpretable (Figure 5a). Gas release was also detected from the CI specimen (Figure 5b); however, the corresponding signals were difficult to analyze because the partial pressures, or gas-release rates, of deuterium-containing species were low and close to the background level.

A qualitative comparison of the integrated deuterium release (Figure 5c) showed that the value obtained for the SIF specimen was approximately 12.4 times higher than that for the CI specimen, whereas the ratio of the corresponding ion fluences was only approximately 3.2. Therefore, the enhanced gas release from the SIF specimen cannot be attributed solely to the larger number of incident deuterium ions. Instead, it indicates more efficient deuterium retention in Be_12_Ti under the SIF irradiation conditions.

The effective retention of deuterium in the SIF specimen was facilitated by the temperature of ion heating and the duration of irradiation. The specimen heating reached 470–480 K. At this temperature, the mobility of embedded deuterium is limited, and the 11-h duration of irradiation provides the continuous formation of vacancies, vacancy clusters, D–V complexes, and other radiation-induced traps. As a result, the retention of deuterium in the damaged area and its subsequent release during TDS analysis were observed.

Unlike the SIF, the heating of the specimen at the first stage of the CI reached 1000 K. The radiation generation of defects was accompanied by their thermal annealing, increased diffusion mobility of deuterium, its release from traps and desorption directly during irradiation. At the second stage of irradiation, after reducing the intensity of the ion flux, the CI specimen temperature decreased to 420 K. The duration of exposure in this mode was only 60 min. In comparison with the 11-h exposure in the SIF mode, 60 min of ion exposure in the second stage of the CI mode was not enough to retain the amount of deuterium comparable to the SIF.

The SIF specimen exhibited multicomponent gas-release behavior. The principal D_2_ release occurred in the intermediate-temperature range, with maxima at approximately 650 and 790 K, with the component centered near 790 K accounting for the largest fraction of the integrated signal. This release may be associated with deuterium detrapping from irradiation-induced trapping sites of intermediate binding energy, including vacancies, vacancy clusters, and D-V complexes. The HD signal exhibited a more complex structure and included high-temperature components at approximately 800 and 1050 K. This finding indicates the presence of deeper and more stable trapping states, which may be associated with defect complexes, dislocation structures, and a spatially extended damaged region produced by high-energy ion irradiation.

The observed distribution of the TDS peaks is consistent with the proposed differences in deuterium behavior under the CI and SIF conditions. During CI, the specimen temperature reached approximately 1000 K, which was sufficiently high to activate deuterium diffusion and promote its release from some trapping sites during irradiation itself. Consequently, the subsequent TDS signal from the CI specimen was relatively weak. By contrast, the SIF specimen was irradiated at a substantially lower temperature, not exceeding approximately 480 K. These conditions restricted deuterium mobility and promoted its accumulation at irradiation-induced defects. During subsequent TDS heating, the trapping sites were progressively emptied, resulting in intense D_2_ and HD release.

The sharp peak observed in the SIF gas-release curve at approximately 630–650 K may be associated not only with the thermal release of deuterium from relatively deep traps but also with mechanical fracture of the specimen during the TDS measurement. The specimen most likely fractured because of internal pressure buildup within the gas-saturated damaged region formed during prolonged D^+^-ion irradiation. Upon crack opening, previously isolated regions containing accumulated deuterium may have become abruptly connected to the specimen surface and the vacuum environment, producing a step-like increase in the partial pressure recorded in the TDS curve. The subsequent gradual decrease in the signal can be interpreted as depletion of the main deuterium inventory accumulated in the SIF specimen after opening of the damaged region and rapid release of a substantial fraction of the retained gas.

Importantly, the blank TDS run showed no significant signals at mass-to-charge ratios of 3 and 4. Therefore, the HD and D_2_ peaks can be attributed specifically to deuterium released from the irradiated Be_12_Ti specimen rather than to outgassing from the vacuum system. This observation supports the conclusion that the SIF specimen accumulated a considerable amount of deuterium within the material bulk. The depth distribution of implanted deuterium, resulting from the high ion energy of 14 MeV and a penetration depth of several hundred micrometers, also explains the broad shape of the TDS peaks. Under these conditions, gas release is governed not only by the binding energies of the trapping sites but also by diffusion-mediated transport of deuterium from the deeper irradiated regions toward the surface.

Thus, the TDS results confirm that deuterium retention in Be_12_Ti is governed primarily by irradiation-induced defects. At least two groups of trapping sites can be distinguished in the SIF specimen: intermediate-energy traps responsible for the major lower- and intermediate-temperature D_2_ and HD release, and deeper, more stable defect states associated with the high-temperature HD components above 800 K. The sharp peak at approximately 630–650 K may indicate the opening of a gas-saturated damaged region and the mechanical fracture of the specimen, whereas the subsequent decrease in the signal may reflect the gradual depletion of the accumulated deuterium inventory. Taken together, these results support the initial hypothesis that the elevated temperature reached during CI promoted the release of a fraction of the implanted deuterium during irradiation, whereas the lower temperature under SIF conditions facilitated its retention within the defect structure until subsequent TDS heating.

**Figure 6:**
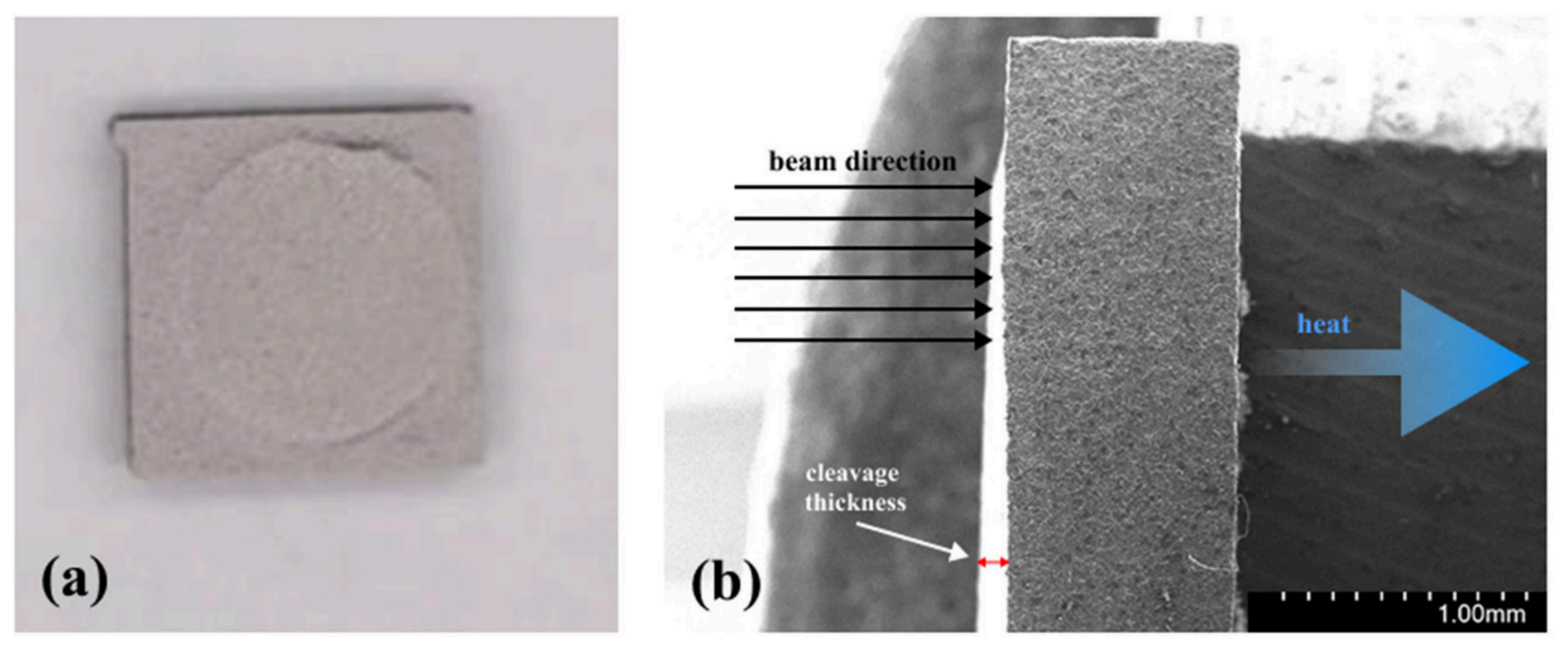
Microscale images of the Be_12_Ti specimen irradiated under the stationary ion flow (SIF) regime: (**a**) front view and (**b**) side view.

Fracture of the specimen was observed upon its removal from the TDS system. Figure 6a shows the fracture surface of a portion of the Be_12_Ti specimen irradiated under the SIF regime after the TDS experiment. A distinct circular boundary is visible in the central part of the specimen, visually separating the central irradiated region from the peripheral region. The central area corresponded to the region exposed to the deuterium-ion beam, in which a high concentration of irradiation-induced defects was generated.

The visibility of the irradiated region after fracture can be attributed to material separation near the depth corresponding to the projected ion range. The crack propagated approximately parallel to the irradiated surface at a depth where the pressure generated by accumulated deuterium exceeded the fracture resistance of the intermetallic matrix.

Figure 6b presents a cross-sectional view of the fractured Be_12_Ti specimen obtained by scanning electron microscopy. The fracture in the peripheral region propagated strictly parallel to the irradiated surface. The thickness of the detached layer was 1.0 ± 0.1 mm. A protruding irradiated region is clearly visible on the left-hand side of the specimen and appears bright because of topographic contrast. The irradiated region is thicker near the center of the specimen and gradually becomes thinner toward the periphery. This variation is presumably associated with the non-uniform ion-flux distribution, with a higher ion flux density at the center of the beam than at its periphery. Consequently, a higher density of irradiation-induced defects and greater internal stresses were generated in the central region. Fracture was initiated near the center of the specimen, after which the crack propagated along the boundary of the highly stressed region.

**Figure 7:**
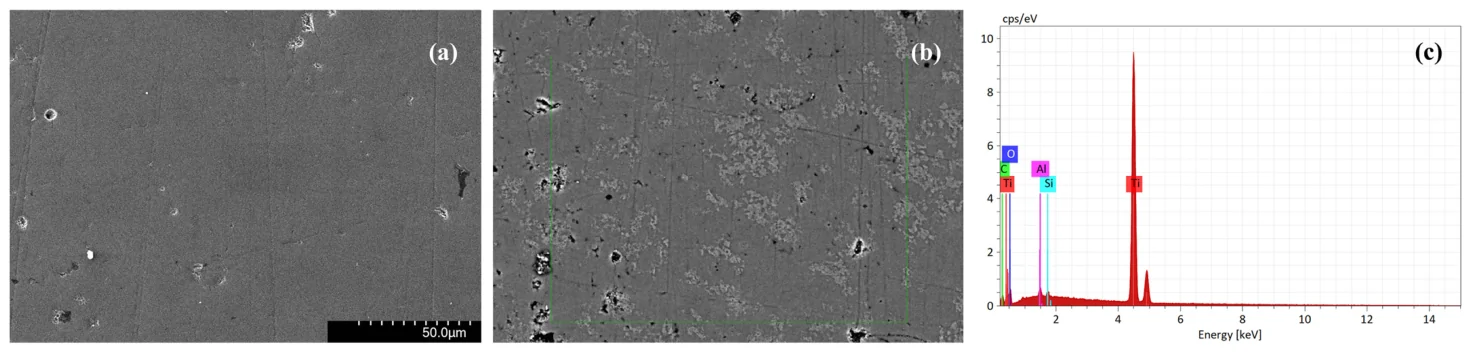
Microstructure and elemental composition of the specimen irradiated under the stationary ion flow (SIF) regime: (**a**) surface after irradiation; (**b**) region selected for EDS analysis; and (**c**) EDS results.

Figure 7 shows surface images of the Be_12_Ti specimen following deuterium-ion irradiation under the SIF regime. As can be seen from Figure 7a,b, SIF did not produce any pronounced changes in the surface morphology. The pre-existing discontinuities and polishing marks remained visible. No clear evidence of surface erosion, blistering, or flaking was observed.

Bright regions were detected on the specimen surface (Figure 7b). Elemental analysis showed that these regions contained oxygen, aluminum, and silicon (Figure 7c), which are constituents or impurities associated with the corundum washer. Their presence may be attributed to ion-beam-induced sputtering of the corundum washer, followed by redeposition of the sputtered material onto the specimen surface.

**Figure 8:**
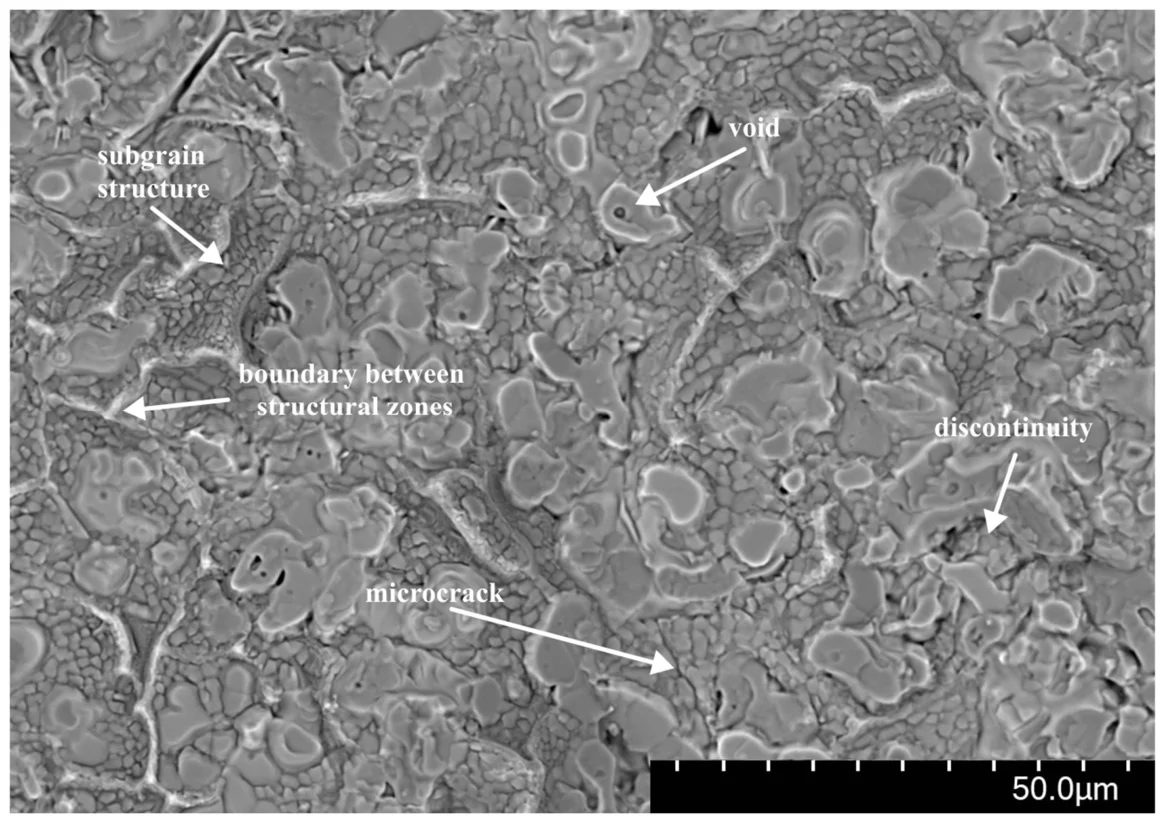
Microstructural region of the specimen exposed to deuterium-ion bombardment under the combined irradiation (CI) regime.

Figure 8 shows the surface of the Be_12_Ti specimen after irradiation under the combined irradiation (CI) regime. The results indicate that intense deuterium-ion bombardment induced both thermal and irradiation-assisted degradation of the Be_12_Ti surface. The material exhibited a polycrystalline microstructure. The characteristic size of the visible microstructural features, corresponding to the initial powder particles, ranged from approximately 10 to 35 µm. The boundaries between the microstructural features were clearly defined and exhibited an irregular, tortuous morphology. In some regions, these boundaries were accentuated by topographic contrast arising from electron scattering at sharp edges and local variations in surface height.

Within individual large particles, a quasi-equiaxed ultrafine subgrain structure with subgrain sizes of up to 1.5 µm was clearly observed. This feature indicates the occurrence of polygonization and recrystallization processes induced by the combined effects of displacement cascades and intense thermal heating to approximately 1000 K. The formation of this ultrafine structure may be attributed to rapid thermally activated recrystallization of the matrix at 1000 K, enhanced by the high density of irradiation-induced defects, including vacancies and dislocation loops, generated by 14 MeV ions.

Localized cracks were observed along particle and grain boundaries. These cracks were most likely caused by thermal stresses and anisotropic thermal expansion of the tetragonal lattice during rapid ion-beam heating in the first irradiation stage and subsequent cooling during the second stage.

Large melt-like agglomerates with rounded, amoeboid, and droplet-like morphologies were also clearly distinguished. Their dimensions reached up to approximately 15 µm, and they exhibited smooth, apparently reflowed edges. The presence of such regions suggests that locally critical temperatures may have been reached. Within some of these melt-like agglomerates, dark, rounded submicrometer features with diameters of approximately 0.2–0.8 μm were observed and may be interpreted as pores. Larger open pores and irregularly shaped cavities, approximately 2–4 µm in size, were also present, for example, in the left and central regions of the micrograph. Their morphology is consistent with the rupture and opening of gas-filled pores.

**Figure 9:**
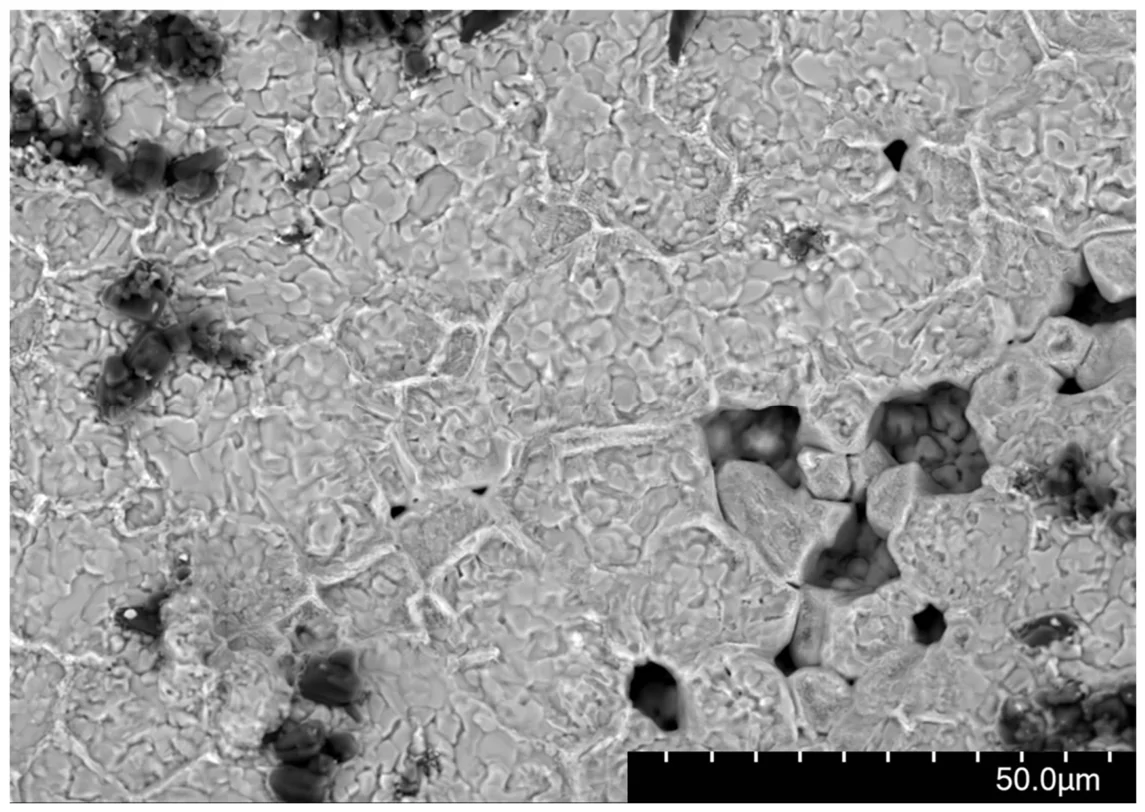
Surface region of the specimen shielded by the corundum washer during irradiation under the combined irradiation (CI) regime.

Figure 9 shows the surface of the Be_12_Ti specimen that was shielded from the ion beam by the corundum washer during irradiation under the CI regime. This region was not directly exposed to ion irradiation. Therefore, the observed surface modifications were caused exclusively by thermal effects resulting from heat conduction from the adjacent irradiated region.

The micrograph clearly reveals the contours of the original polyhedral microstructural constituents, corresponding to the initial powder particles, with sizes ranging from approximately 25 to 40 µm. The particle boundaries appear as bright relief-like lines. This feature may be attributed to thermal etching at crystallite junctions during exposure to 1000 K under vacuum conditions.

The interior of the particles is subdivided into quasi-equiaxed subgrains with sizes of approximately 2–4 µm. Because this region was not directly exposed to the ion beam, the observed mosaic-like substructure can be attributed to thermal polygonization. This process involves the relaxation of internal growth stresses within the intermetallic compound through the rearrangement of dislocations into low-angle boundaries at elevated temperature.

Deep, irregularly shaped discontinuities with dimensions of approximately 10–25 μm are visible in the lower-right region of the micrograph. These discontinuities appear to have formed as a result of intergranular spallation, as indicated by the exposed subgrain fragments with smoothed edges within the cavities. The smooth and faceted morphology of the subgrains inside these discontinuities is also indicative of high-temperature exposure.

Dark regions are observed in the upper-left corner and locally across the specimen surface. These regions correspond to a carbon- and oxygen-containing deposit. Its formation may be associated with the thermal decomposition and subsequent deposition of carbon- and oxygen-containing residual species on the heated surface.

It must be noted that the research methods used in this research (SEM, XRD) are limited for a detailed study of the nature of the identified structural and phase changes in material structure. A more detailed study of the mechanisms of radiation-induced release of secondary phases and segregation of elements in nanoscale will be carried out in future studies of individual “Cross-section regions” using TEM and SAED methods. Unfortunately, the use of TEM and SAED was not possible within the framework of this project.

## 4. Conclusions

The obtained results demonstrate fundamentally different mechanisms of structural and phase degradation, as well as distinct hydrogen-isotope sorption kinetics in the Be_12_Ti intermetallic compound, depending on the severity of the irradiation conditions. Stationary ion flow resulted predominantly in disordering of the crystal structure, whereas the more severe combined irradiation regime promoted thermodynamically favorable phase-separation processes. The identified BeO polymorphs and TiC phase indicate the occurrence of irradiation-assisted oxidation and carbide-formation reactions on freshly generated surfaces.

The multicomponent nature of the TDS spectra obtained for the specimens irradiated under the SIF regime indicates the sequential thermal activation of different deuterium-trapping sites. The presence of high-temperature desorption components demonstrates that irradiation with high-energy 14 MeV ions produces not only simple point defects, such as vacancies, but also deeper and more stable trapping sites, including dislocation structures, vacancy clusters, and spatially distributed damaged regions within the material bulk.

At the same time, the high ion flux density applied during the first stage of the CI regime caused intense heating of the material to approximately 1000 K. At this temperature, the available thermal energy is sufficient to overcome the activation barriers associated with most intermediate-energy defect traps. Consequently, in situ deuterium desorption occurred, with a substantial fraction of the implanted deuterium migrating toward the surface and leaving the material during irradiation. This behavior is consistent with the fundamental ability of beryllide materials to facilitate tritium release at blanket operating temperatures.

The developed simulation-testing methodology based on 14 MeV light deuterium-ion beams provides an effective experimental approach for investigating bulk gas accumulation and diffusion processes while simultaneously combining irradiation-induced defect generation with intense thermal loading. The proposed methodology reproduces selected operating scenarios relevant to fusion reactors, including steady-state plasma operation and abrupt transient events such as plasma disruptions and edge-localized modes.

## Figures and Tables

**Table 1:** D^+^ ion irradiation parameters for both regimes.

	Combined Irradiation (CI)			Stationary Ion Flow (SIF)
Irradiation parameters	20 µA in 22 min	2 µA in 60 min	Σ for CI	2.7 µA in 11 h
Fluence, [ions·cm^−2^]	1.02 × 10^18^	0.283 × 10^18^	1.303 × 10^18^	4.2 × 10^18^
Flux density, [ions·cm^−2^·s^−1^]	7.84 × 10^14^	0.786 × 10^14^	–	1.06 × 10^14^

## Data Availability

The original contributions presented in this study are included in the article. Further inquiries can be directed to the corresponding author.
